# *Poaceae* vs. Abiotic Stress: Focus on Drought and Salt Stress, Recent Insights and Perspectives

**DOI:** 10.3389/fpls.2017.01214

**Published:** 2017-07-11

**Authors:** Simone Landi, Jean-Francois Hausman, Gea Guerriero, Sergio Esposito

**Affiliations:** ^1^Dipartimento di Biologia, Università di Napoli “Federico II” Napoli, Italy; ^2^Environmental Research and Innovation Department, Luxembourg Institute of Science and Technology Esch-sur-Alzette, Luxembourg

**Keywords:** drought stress, salinity, G6PDH, cell wall, silicon, *Hordeum vulgare*, *Triticum aestivum*, *Oryza sativa*

## Abstract

*Poaceae* represent the most important group of crops susceptible to abiotic stress. This large family of monocotyledonous plants, commonly known as grasses, counts several important cultivated species, namely wheat (*Triticum aestivum*), rice (*Oryza sativa*), maize (*Zea mays*), and barley (*Hordeum vulgare*). These crops, notably, show different behaviors under abiotic stress conditions: wheat and rice are considered sensitive, showing serious yield reduction upon water scarcity and soil salinity, while barley presents a natural drought and salt tolerance. During the green revolution (1940–1960), cereal breeding was very successful in developing high-yield crops varieties; however, these cultivars were maximized for highest yield under optimal conditions, and did not present suitable traits for tolerance under unfavorable conditions. The improvement of crop abiotic stress tolerance requires a deep knowledge of the phenomena underlying tolerance, to devise novel approaches and decipher the key components of agricultural production systems. Approaches to improve food production combining both enhanced water use efficiency (WUE) and acceptable yields are critical to create a sustainable agriculture in the future. This paper analyzes the latest results on abiotic stress tolerance in *Poaceae*. In particular, the focus will be directed toward various aspects of water deprivation and salinity response efficiency in *Poaceae*. Aspects related to cell wall metabolism will be covered, given the importance of the plant cell wall in sensing environmental constraints and in mediating a response; the role of silicon (Si), an important element for monocots' normal growth and development, will also be discussed, since it activates a broad-spectrum response to different exogenous stresses. Perspectives valorizing studies on landraces conclude the survey, as they help identify key traits for breeding purposes.

## Introduction

One of the most impelling global challenges is the provision of enough food worldwide. The Food and Agriculture Organization (FAO) has indeed estimated a dramatic increase in need for food by 2050 (Cobb et al., 2013). Agriculture represents the main source of global food and plant breeders need to discern the potential improvement of traits in order to increase the yields (Tester and Langridge, 2010). The urgent request of an increase in food production is not easy to achieve even in a stable and optimal agricultural environment; moreover, this scenario is rapidly worsening, because of different factors, such as, the increase of land- and water-use for biofuel-crops, climate change and abiotic stresses (Ruggiero et al., 2017).

Abiotic stresses induce a wide range of molecular, biochemical and physiological alterations in plants, including enhanced accumulation of osmolytes, reduced photosynthesis, stomata closure and the induction of stress-responsive genes (Yamaguchi-Shinozaki and Shinozaki, 2006; Lata et al., 2015).

Water scarcity and soil salinity undoubtedly represent the major limiting factors for plant cultivation and crop productivity (Hayashi and Murata, 1998; Reynolds and Tuberosa, 2008), as they trigger oxidative, osmotic and temperature stresses. Drought, heat and salt stress cause dehydration, which in its turn results in cytosolic and vacuolar volume reduction (Bartels and Sunkar, 2005). Reactive oxygen species (ROS) are also produced, which provoke damages to proteins, DNA and membranes (Gill and Tuteja, 2010). Some of the most critical further damages induced by these stresses are: reduction in photosynthesis rate and efficiency, wilting and induction of programmed death cell.

Many other factors play a considerable role in drought and salt stress responses, e.g., SOS (Salt Overly Sensitive) pathway, kinases, phosphatases, abscisic acid (ABA), ion transporters, transcription factors (Lata and Prasad, 2011; Lata et al., 2011), but they will not be discussed in detail in this review, as they have been already extensively treated in previous works.

The role of *Poaceae* in worldwide food demand is critical: rice is the most important food source for more than half of the world population, (Cui Y. et al., 2016) wheat provides nearly 55% of carbohydrates worldwide (Gill et al., 2004), barley is the fourth most important cereal crop in terms of planting area, mainly used in brewing industries and as forage (Shen et al., 2016).

The natural resistance of barley to exogenous stresses makes it the most tolerant among *Poaceae* and an important model in stress physiology (Gürel et al., 2016; Shen et al., 2016). Nonetheless, several high-yield *H. vulgare* cultivars have become sensitive to abiotic stress by loss of genetic variation induced by breeding programs; hence, the response to exogenous constraints has become an important issue in barley as well (Ahmed et al., 2013).

Climate changes between 1980 and 2008 caused a significant yield loss in different crops, including *Poaceae*: rice showed a remarkable yield reduction in China and in developing countries; the global production of maize and wheat decreased by 3.8 and 5.5%, respectively (Lobell et al., 2011). These data highlight the importance of studies addressing stress physiology in *Poaceae* and the need to conceive strategies improving specific traits under unfavorable conditions for these economically important crops.

In the present study we will provide an overview of the latest advances in the field of *Poaceae* stress physiology by focusing, specifically, on drought and salt stress.

### Stress-responsive genes in *Poaceae*: insights from ROS scavengers and water use efficiency-related genes

One of the key mechanisms increasing the adaptation to adverse environmental conditions in plants is the regulation of the reactive oxygen species (ROS) levels (Gill and Tuteja, 2010).

Recently, several abiotic stress-related genes conferring tolerance in *Poaceae* have been described (key representatives are summarized in Table 1).

**Table 1:** List of genes conferring abiotic stress tolerance in *Poaceae*.

**Gene**	**Annotation**	**Function**	**Stress**	**Overexpression/inactivation**	**Species**	**References**
*G6PDH*	Glucose-6P dehydrogenase	NADPH provision	Salt	Overexpression	*H. vulgare*	Cardi et al., 2015
*HVA1*	LEA protein	Enhanced water use efficiency	Drought	Overexpression	*H. vulgare*	Gürel et al., 2016
*HvPIP2;5*	Plasma membrane intrinsic protein 2;5	Acquaporin	Salt and Osmotic	Overexpression	*H. vulgare*	Alavilli et al., 2016
*OsNAC9*	NAC domain containing protein 9	Enhanced Uptake in roots	Drought	Overexpression	*O. sativa*	Redillas et al., 2012
*OsASR5*	Abscissic acid and ripening protein 5	Regulation of stomatal closure and chaperon-like protein	Drought	Overexpression	*O. sativa*	Li et al., 2016
*OsJRL*	Jacalin-related lectin	Cell protection and signal transduction	Salt	Overexpression	*O. sativa*	He X. et al., 2016
*OsMYB55*	MYB transcription factor 55	Modulating expression of drought related genes	Drought	Overexpression	*O. sativa*	Casaretto et al., 2016
*OsNAC2*	NAC domain containing protein 2	Reduction of LEA and SAPK expression	Drought	Inactivation	*O. sativa*	Shen et al., 2017
*OsPEX11*	Peroxisomal biogenesis factor 11	Regulation in Na^+^/K^+^ transporter, reduction in ROS accumulation	Salt	Overexpression	*O. sativa*	Cui P. et al., 2016
*OsSAP1*	Stress associated protein 1	Increase root growth and fresh weight	Drought and Salt	Overexpression	*O. sativa*	Kothari et al., 2016
*OsSGL*	Stress tolerance and grain length protein	Increase osmolytes accumulation and root growth	Drought	Overexpression	*O. sativa*	Cui Y. et al., 2016
*OsVPE3*	Vacuolar processing enzyme 3	Vacuole-mediated PCD, Stomatal development	Salt	Inactivation	*O. sativa*	Lu et al., 2016
*TaCRT1*	Calreticulin	Enhanced ROS scavenging activation	Salt	Overexpression	*T. aestivum*	Xiang et al., 2015
*TaGBF1*	G-box binding factor	Induced hypersensivity to salt stress	Salt	Inactivation	*T. aestivum*	Sun et al., 2015
*TaFER-5B*	Ferritin	Reduction in ROS accumulation	Drought and Heat	Overexpression	*T. aestivum*	Zang et al., 2017
*TaMYB31*	MYB transcription factor 31	Cuticle synthesis	Drought	Overexpression	*T. aestivum*	Bi et al., 2016
*TaMYB74*	MYB transcription factor 74	Cuticle synthesis	Drought	Overexpression	*T. aestivum*	Bi et al., 2016
*TaWRKY1*	WRKY transcription factor 1	Promoted root growth	Drought	Overexpression	*T. aestivum*	He G. H. et al., 2016
*TaWRKY33*	WRKY transcription factor 33	Promoted root growth	Drought	Overexpression	*T. aestivum*	He G. H. et al., 2016
*TdMnSOD*	Superoxide dismutase	Enhanced ROS scavenging action	Drought and Salt	Overexpression	*T. turgidum*	Kaouthar et al., 2016
*ZmABA2*	Dehydrogenase/ reductase SDR	Regulation of ABA content	Drought	Overexpression	*Z. mays*	Ma F. et al., 2016
*ZmGOLS2*	Galactinol synthase	Raffinose synthesis	Drought and Salt	Overexpression	*Z. mays*	Gu et al., 2016
*ZmMPK5*	MAP kinase 5	Regulation of ABA content	Drought	Overexpression	*Z. mays*	Ma F. et al., 2016
*ZmPP2C-A10*	Protein phosphatase 2C	Negative controller of ABA pathway	Drought	Inactivation	*Z. mays*	Xiang et al., 2016

*The gene names, annotation, function, type of stress, species and references are indicated. Overexpression or inactivation of the specific genes conferring tolerance are indicated*.

The manganese-dependent superoxide dismutase (Mn-SOD) of *Triticum turgidum* (*Td*MnSOD) expressed in *Arabidopsis thaliana* enhanced the tolerance to multiple abiotic stresses by promoting proline accumulation and lowering H_2_O_2_ content (Kaouthar et al., 2016). Similarly, tobacco overexpressing the *T. aestivum* calreticulin protein 1 (CRT1, which plays important roles in Ca^2+^ signaling and protein folding) showed an enhanced salt tolerance with respect to wild type plants (Xiang et al., 2015).

Reactive oxygen species (ROS) must be regulated by enhancing ROS scavenging and/or reinforcing pathways preventing their dangerous increase. In this respect, ferritin gene expression is known to be induced in response to drought, salinity and other stresses (Ravet et al., 2009): *Arabidopsis* ferritin genes were indeed upregulated by H_2_O_2_, iron and ABA. Zang et al. (2017) recently described the interesting potential of *T. aestivum* ferritin: *A. thaliana* plants transformed with *Ta*FER-5B and overexpressing transgenic wheat plants showed a lower accumulation of ${\text{O}}_{2}^{-}$ and H_2_O_2_, resulting in enhanced heat and drought stress tolerance. These results also demonstrate that monocot genes can confer increased resistance to stresses when heterologously expressed in dicots.

Significant improvements in abiotic stress tolerance by ROS detoxification were obtained also in rice (*Oryza sativa—Os). Os*SGL (Stress tolerance and Grain Length) codes for a putative DUF1645 domain-containing protein: *A. thaliana* plants transformed with *Os*SGL and overexpressing rice plants showed enhanced drought and osmotic stress tolerance (Cui Y. et al., 2016). Additionally, RNA-Seq on rice plants overexpressing *Os*SGL highlighted an increase in expression of several stress-responsive genes; among these, a number of peroxidases were identified, thereby correlating the *Os*SGL action with an enhanced ROS scavenging system.

More recently, we have suggested an emerging role in salt and drought stress response for glucose-6-phosphate dehydrogenase (G6PDH–EC 1.1.1.49) (Cardi et al., 2015; Esposito, 2016; Landi et al., 2016). This enzyme catalyzes the oxidation of glucose-6-phosphate (G6P) and the corresponding reduction of NADP^+^ to NADPH. The increase in G6PDH activity in barley upon salt stress resulted in an increased NADPH production, able to confer stress resistance through the synthesis of osmolytes (e.g., glycine betaine) and ROS scavengers, such as, glutathione (Cardi et al., 2015). This response, notably, is linked to an ABA-dependent pathway (Cardi et al., 2011; Dal Santo et al., 2012).

Another important parameter in the response of crops to dehydration and osmotic imbalances is Water Use Efficiency (WUE). This condition is connected with a decreased photosynthetic rate, plant growth and productivity; usually higher WUE values result in lower stomata conductance (Ruggiero et al., 2017).

Root architecture plays a critical role in WUE in cereals and this is controlled by a number of transcription factors (TFs). The overexpression of the NAC-domain-containing TF *Os*NAC9 induced drought tolerance in rice transgenic plants, and triggered the formation of an enlarged stele and aerenchyma (Redillas et al., 2012). WRKY is another class of TFs inducing drought tolerance by root modification. The complete WRKY set of *T. aestivum* has been described (He G. H. et al., 2016): among them, *Ta*WRKY1 and *Ta*WRKY33 were identified as drought-related factors, and used to induce tolerance to *Arabidopsis* transgenic lines by promoting germination and root growth.

An increased WUE can also be obtained by stimulating wax deposition in the cuticle. In this respect, promising evidences in wheat have been recently obtained using *Ta*MYB31 and *Ta*MYB74 (Bi et al., 2016), two drought-responsive TFs involved in cuticle biosynthesis. When overexpressed transiently in wheat cells using particle bombardment, these TFs were able to activate the promoters of those genes involved in cuticle biosynthesis (Bi et al., 2016): therefore these transcriptional regulators can be used to increase WUE in monocots under drought.

TFs, particularly master regulators, are able to modulate entire pathways (clear examples come from TFs involved in secondary cell wall or suberin deposition; Nakano et al., 2015; Legay et al., 2016), therefore they represent useful candidates for more efficient biotechnological strategies.

ROS scavenging and WUE efficiency are connected by a number of ABA-related genes involved in transduction and transcription pathways. Examples of genes inducing abiotic stress tolerance in *Poaceae* are *Os*ASR5, *Zm*ASR1, *Zm*ABA2, *Zm*MPK5: it was demonstrated that these genes connect the regulation of stomatal closure with the antioxidant response and ABA signaling (Virlouvet et al., 2011; Li et al., 2016; Ma F. et al., 2016).

Furthermore, in *Poaceae*, specific TFs and water channels play a crucial role in the response to abiotic stress: transgenic maize overexpressing the *Zm*MYB55 showed an increase in drought and heat stress tolerance by reducing ROS levels and lipid peroxidation (Casaretto et al., 2016). Similarly, *A. thaliana* plants overexpressing the *H. vulgare* aquaporin *Hv*PIP2; 5 (PIP: Plasma-membrane Intrinsic Protein) revealed an improved tolerance to salt, drought and osmotic stresses (Alavilli et al., 2016). Intriguingly, these genes were co-expressed together with ROS antioxidant enzymes as SOD, catalase (CAT) and ascorbate peroxidase (APX).

### The role of cell wall-related genes in *Poaceae* stress physiology

Cell wall is a natural biocomposite of polysaccharides, proteins and, in certain cases, of the aromatic macromolecule lignin (Guerriero et al., 2014b, 2016b). It is an active structure which partakes in crucial stages of plant development and in cell signaling under exogenous stresses (Parrotta et al., 2015). A specific system of sensors ensures indeed the perception of the cell wall integrity status, which becomes perturbed upon i.e. pathogen attack or abiotic stresses (Gall et al., 2015; Hamann, 2015). Besides the cell wall integrity status perception, plants modulate cell wall-related processes in the presence of exogenous constraints (Guerriero et al., 2014a; Behr et al., 2015): an emblematical example is the deposition of stress lignins upon the sensing of exterior constraints (Moura et al., 2010). Nowadays, thanks to next generation sequencing (NGS), huge dataset are generated which can help shed light on the dynamics of cell wall-related genes in different species. In this paragraph we provide an example using *O. sativa* as a model, for which the entire genome sequence is available (International Rice Genome Sequencing Project, 2005). We used the rice expression database at http://rice.plantbiology.msu.edu/expression.shtml, in order to get an overview of salt and drought responsive cell wall-related genes in this important crop (Ouyang et al., 2007). Drought and salt stress treatments in rice (cv. *Nipponbare*) induced 551 and 1,600 genes in leaves and roots, respectively. In the stress-repressed category, 323 and 613 genes were observed in leaves and roots, respectively. Among these, a significant number of genes belonging to the GO categories “cell wall” (GO: 005618) was found (details are in Table 2). Specifically, 13 and 31 stress-induced and 2 and 41 stress-repressed genes were found in roots and leaves, respectively. Therefore, cell wall remodeling represents a common mechanism in abiotic stress response: plant cell walls are often modified upon drought and salt stress (Tenhaken, 2015). Indeed, wheat cultivars differing in drought stress tolerance were shown to display a different regulation of cell wall-related processes (Leucci et al., 2008).

**Table 2:** List of drought- and salt-responsive genes in *O. sativa*.

**Gene**	**Induced**	**Type of stress**
**LEAVES**		
LOC_Os05g25640.1	*Cytochrome P450*	Drought and Salt
LOC_Os01g11010.1	*Peptide-N4-asparagine amidase A*	Drought
LOC_Os01g11760.1	*GDSL-like lipase/acylhydrolase*	Drought
LOC_Os01g33420.1	*Glycosyl hydrolase family protein 27*	Drought
LOC_Os11g06390.1	*Actin*	Drought
LOC_Os11g29190.1	*40S Ribosomal protein S5*	Drought
LOC_Os02g09940.1	*Peroxiredoxin*	Drought
LOC_Os04g46390.2	*Chaperone protein dnaJ*	Drought
LOC_Os08g02230.1	*FAD-binding and arabino-lactone oxidase domains containing protein*	Drought
LOC_Os11g47760.1	*DnaK family protein*	Salt
LOC_Os12g38170.1	*Osmotin*	Salt
LOC_Os04g03796.1	*OsSub37 - Subtilisin homolog*	Salt
LOC_Os07g38760.1	*HEAT repeat family protein*	Salt
**ROOTS**		
LOC_Os01g07910.1	*NADH-cytochrome b5 reductase*	Drought
LOC_Os01g10950.1	*Peptide-N4-asparagine amidase A*	Drought
LOC_Os01g71670.1	*Glycosyl hydrolases family 17*	Drought
LOC_Os11g24450.1	*Mitochondrial carrier protein*	Drought
LOC_Os12g05040.6	*Heavy-metal-associated domain-containing protein*	Drought
LOC_Os12g12850.1	*ATP-dependent Clp protease ATP-binding subunit clpA homolog*	Drought
LOC_Os02g02410.1	*DnaK family protein*	Drought
LOC_Os02g02400.1	*Catalase isozyme A*	Drought
LOC_Os02g18650.1	*Pectinesterase*	Drought
LOC_Os02g44590.1	*OsSub20 - Subtilisin homolog*	Drought
LOC_Os03g11410.1	*MItochondrial-processing peptidase subunit*	Drought
LOC_Os03g15690.1	*Phosphate carrier protein mitochondrial precursor*	Drought
LOC_Os03g15020.1	*Beta-galactosidase precursor*	Drought
LOC_Os03g49600.1	*Os3bglu7 - beta-glucosidase exo-beta-glucanase*	Drought
LOC_Os03g55110.1	*26S proteasome non-ATPase regulatory subunit 10*	Drought
LOC_Os04g41740.1	*Expressed protein*	Drought
LOC_Os04g44410.1	*OsSCP25 - Serine Carboxypeptidase homolog*	Drought
LOC_Os04g46390.2	*Chaperone protein dnaJ*	Drought
LOC_Os04g56930.1	*Glycosyl hydrolases GH32*	Drought
LOC_Os05g29790.1	*Pectinesterase*	Drought
LOC_Os06g40600.1	*Elongation factor*	Drought
LOC_Os06g48650.2	*OsSub52 - Subtilisin homolog*	Drought
LOC_Os07g38730.1	*Tubulin/FtsZ domain containing protein*	Drought
LOC_Os07g44780.1	*GDSL-like lipase/acylhydrolase*	Drought
LOC_Os08g39500.1	*60S ribosomal protein L31*	Drought
LOC_Os08g39140.1	*Heat shock protein*	Drought
LOC_Os01g22010.3	*S-adenosylmethionine synthetase*	Salt
LOC_Os10g33140.1	*hcrVf2 protein*	Salt
LOC_Os11g03400.1	*Ribosomal protein*	Salt
LOC_Os02g10070.2	*Citrate synthase*	Salt
LOC_Os09g25910.1	*Xylanase inhibitor*	Salt
**Gene**	**Repressed**	**Type of stress**
**LEAVES**		
LOC_Os12g02310.1	*LTPL11 - Protease inhibitor/seed storage/LTP family*	Drought and Salt
LOC_Os07g04240.1	*Succinate dehydrogenase flavoprotein*	Drought and Salt
**ROOTS**		
LOC_Os01g01060.1	*40S Ribosomal protein S5*	Drought and Salt
LOC_Os01g05490.1	*Triosephosphate isomerase cytosolic*	Drought and Salt
LOC_Os01g18170.1	*Cupin domain containing protein*	Drought and Salt
LOC_Os01g28450.1	*SCP-like extracellular protein*	Drought and Salt
LOC_Os01g54620.1	*CESA4 - cellulose synthase*	Drought and Salt
LOC_Os01g67240.1	*Formin-like protein 1 precursor*	Drought and Salt
LOC_Os01g68324.3	*Dolichyl-diphosphooligosaccharide–protein glycosyl transferase 63 kDa*	Drought and Salt
LOC_Os01g71060.1	*Xylanase inhibitor*	Drought and Salt
LOC_Os10g11500.1	*SCP-like extracellular protein*	Drought and Salt
LOC_Os10g25400.1	*GDSL-like lipase/acylhydrolase*	Drought and Salt
LOC_Os10g26680.1	*Pectinesterase*	Drought and Salt
LOC_Os11g06390.1	*Actin*	Drought and Salt
LOC_Os12g21798.1	*40S ribosomal protein S3a*	Drought and Salt
LOC_Os12g32986.1	*Heat shock protein*	Drought and Salt
LOC_Os12g38180.1	*Heat shock cognate 70 kDa protein 2*	Drought and Salt
LOC_Os02g18550.1	*40S ribosomal protein S3a*	Drought and Salt
LOC_Os02g44490.1	*Anthranilate phosphoribosyltransferase*	Drought and Salt
LOC_Os02g51970.1	*Phosphate-induced protein 1 conserved region domain*	Drought and Salt
LOC_Os03g10340.1	*40S ribosomal protein S3a*	Drought and Salt
LOC_Os03g16860.1	*DnaK family protein*	Drought and Salt
LOC_Os03g31510.1	*Cysteine proteinase inhibitor 8 precursor*	Drought and Salt
LOC_Os03g51440.1	*LRR receptor-like protein kinase*	Drought and Salt
LOC_Os03g51600.1	*Tubulin/FtsZ domain containing protein*	Drought and Salt
LOC_Os03g53800.3	*Periplasmic beta-glucosidase precursor*	Drought and Salt
LOC_Os04g24220.1	*OsWAK32 - OsWAK receptor-like protein kinase*	Drought and Salt
LOC_Os04g35090.1	*40S ribosomal protein S10*	Drought and Salt
LOC_Os04g49690.1	*FERONIA receptor-like kinase*	Drought and Salt
LOC_Os04g51460.1	*Glycosyl hydrolases family 16*	Drought and Salt
LOC_Os04g59260.1	*Peroxidase precursor*	Drought and Salt
LOC_Os05g04470.1	*Peroxidase precursor*	Drought and Salt
LOC_Os05g27940.1	*40S ribosomal protein S7*	Drought and Salt
LOC_Os05g32110.1	*COBRA*	Drought and Salt
LOC_Os05g45950.1	*Outer mitochondrial membrane porin*	Drought and Salt
LOC_Os05g48900.1	*Fasc|Iclin domain containing protein*	Drought and Salt
LOC_Os07g04240.1	*Succinate dehydrogenase flavoprotein subunit mitochondrial precursor*	Drought and Salt
LOC_Os07g48030.1	*Peroxidase precursor*	Drought and Salt
LOC_Os07g48060.1	*Peroxidase precursor*	Drought and Salt
LOC_Os08g23180.1	*Fasciclin-like arabinogalactan protein 8 precursor*	Drought and Salt
LOC_Os08g37840.1	*Phosphate-induced protein 1 conserved region domain containing prot*.	Drought and Salt
LOC_Os09g26920.1	*OsSub57 - Subtilisin homolog*	Drought and Salt
LOC_Os09g31486.1	*DnaK family protein*	Drought and Salt

Particularly interesting is the presence of cell wall receptors among the class of genes downregulated in rice roots (Table 2): the downregulation of *OsWAK32* and the ortholog of *FERONIA* is likely related with the inhibition in root elongation observed upon drought/salt stress in rice.

A number of peroxidase (LOC_Os04g59260.1, LOC_Os05g04470.1, LOC_Os07g48030.1, LOC_Os07g48060.1 e.g.,) were found as repressed in roots of rice (Table 2). Cell wall peroxidases play contradicting roles in abiotic stress response. As example, drought tolerant wheat cultivars exposed to osmotic stress showed higher transcripts levels of the peroxidases *TaPrx01, TaPrx03, TaPrx04* (Csiszár et al., 2012). On the other hand, an increase in peroxidase activity may induce the excess in OH^−^ levels, inducing cell wall loosening (Tenhaken, 2015), thus suggesting that a correct balance of peroxidase activities is desirable in abiotic stress tolerance.

Another interesting class of cell wall-related genes regulated by drought and salt stress is represented by pectinesterases (LOC_Os02g18650.1; LOC_Os10g26680.1 e.g.,). Various crops such as, wheat, soybean, tomato, showed higher levels of pectin remodeling enzymes in tolerant cultivars than susceptible genotypes (Leucci et al., 2008; An et al., 2014; Iovieno et al., 2016).

In this respect it should be noted that a study centered on rice identified the presence of methyl esterified pectins in both primary and secondary cell walls and the differential expression of pectin methylesterases upon stress treatments (Jeong et al., 2015).

In rice the role of genes encoding both hydrolytic enzymes and glycosyltransferases can be analyzed via publicly available databases, notably the Rice GH and GT databases (Cao et al., 2008, 2010; Sharma et al., 2013).

Notably, GHs represent an important group of cell-wall related enzymes involved in the remodeling of the cell wall structure, particularly under stress: as expected, at least two GH genes are activated under drought (LOC_Os01g71670.1; LOC_Os04g56930.1) in rice roots, while another gene (LOC_Os04g51460.1) is repressed by drought and salt stress (Table 2).

The expression pattern of cell wall-related genes in rice, as well as other monocots, helps in the identification of specific trends in response to exogenous stresses shared between the dicot and monocot lineages. Houston et al. (2016) made a detailed survey of the microarray data available for *Arabidopsis* and barley and highlighted those genes coding for CAZymes (Carbohydrate-Active Enzymes) upregulated in both *Arabidopsis* and barley in response to biotic and abiotic stress. The analyses revealed members of families GT1, GT8, GT61, GT75 (the role of GTs in response to exogenous stresses is less understood than that of GHs) as being upregulated in both organisms in response to the stresses.

### Silicon: a crucial element for *Poaceae* and a stress reliever

Silicon (Si) is an abundant element in soils and it plays an important role in plants, improving vigor, productivity and stress resistance. Si (taken up by plants as silicic acid) acts as a priming agent and activates the defense response arsenal of plants, by stimulating the metabolism, both primary and secondary, via still not fully understood mechanisms (recently reviewed by Guerriero et al., 2016a). Since among *Poaceae* there are representatives classified as high Si-accumulators (notably the commelinoid monocot rice, which can accumulate up to 10% of the shoot dry weight; Ma et al., 2002) and given its role in boosting the plant response to exogenous stresses, we believe it necessary to comment on its role in *Poaceae* abiotic stress response.

In monocots, like rice, Si is considered essential (Savant et al., 1997), despite the current classification of this element as quasi-essential (Epstein and Bloom, 2005), because its absence triggers dramatic consequences, namely yield loss, pathogen and abiotic stress increased susceptibility (Datnoff and Rodrigues, 2005). This importance may be partly due to the nature of monocots' cell walls, which are of type II (Yokoyama and Nishitani, 2004) and where Si may play a structural role connected to cell wall integrity, in a manner analogous to B in dicots' type I cell walls.

Si was shown to protect barley under Cr stress: it alleviated the ultrastructural disorders in both leaves and root tips and ameliorated net photosynthetic rate and stomatal conductance under heavy metal stress (Ali et al., 2013).

The deleterious effects of drought were mitigated by exogenous Si application in wheat: plants treated with Si displayed higher activities of SOD, CAT, and GR (glutathione reductase), had higher amounts of photosynthetic pigments and total thiols, while H_2_O_2_ content and protein oxidative stress decreased (Gong et al., 2005). Another study on wheat, in which gene expression analysis was performed, revealed that the drought tolerance in Si-treated wheat was accompanied by transcriptional reprogramming: genes involved in the ascorbate-reduced glutathione cycle, flavonoid biosynthesis and antioxidant response showed increased expression in Si-supplied plants (Ma D. et al., 2016).

Seed priming with Si can be very effective in protecting the growing plantlets from exogenous stresses: for example maize primed with Si displayed better resistance to alkaline stress through enhanced growth, photosynthesis, leaf relative water content and by increasing the activities of antioxidant enzymes, soluble sugars and proteins, while decreasing the contents of malondialdehyde, proline and Na^+^ (Abdel Latef and Tran, 2016).

Si can also establish positive interactions and synergy with other elements, such as, N: a recent study on rice showed indeed that Si basal application, coupled to post-flood N application, resulted in the highest tolerance to submergence, by reducing lodging, leaf chlorosis and senescence (Gautam et al., 2016).

### Perspectives on the study of abiotic stress tolerance in *Poaceae*: the importance of landraces and their wild relatives

A serious consequence of the modern breeding strategies is the decrease of the agricultural biodiversity; this leads to a reduction in abiotic stress tolerance, because of the loss of specific genomic traits during domestication (Dwivedi et al., 2016). Therefore, the exploitation of the genetic resources represented by local landraces, or wild relatives, is a promising approach to find interesting genetic traits useful to improve breeding strategies of crops. This strategy has already been successfully tested in different *Poaceae* such as, wheat, rice, maize, and barley (Lopes et al., 2015; Dwivedi et al., 2016; Van Oosten et al., 2016). As example, different wheat and rice landraces have been recently used in high-throughput studies, describing thousands of SNPs possibly able to enhance drought and salt tolerance (Huang et al., 2010; Cavanagh et al., 2013). Moreover, at least 22 wild relatives of *Oryza* are known, and their genetic diversity can provide novel traits for drought tolerance (Van Oosten et al., 2016). Furthermore, wild relatives of rice (*Oryza rufipogon* Griff.), were recently used for a miRNA sequencing in drought stress condition (Zhang et al., 2017). The study led to the identification of 162 known miRNAs differentially expressed in drought stress condition and, notably, 69 new miRNAs candidates.

Recently, the genome sequencing of the Tibetan barley (*Hordeum vulgare* L. var. *nudum*), highlighted an interesting expansion of stress-related gene families (Zeng et al., 2015).

In recent years, new introgression lines between commercial cultivars and wild relatives have been generated in *Poaceae*. As an example, the wild rice Dongxiang accession (*O. rufipogon* Griff.) was used to generate drought tolerant accessions by the introgression of genetic traits in modern rice cultivars (Zhang et al., 2006).

Significant results in abiotic stress tolerance traits introgression were obtained also in *Triticum*. Wheat was improved in drought and salt stress tolerance by the wild relatives *Aegilops umbellulata* and *Agropyron elongatum* (Molnar et al., 2004; Colmer et al., 2006; Placido et al., 2013).

More recently, interesting abiotic stress-related loci were described in wild barley. Shen et al. (2016) showed a high salt tolerance in Tibetan wild barley (*Hordeum spontaneum* C. Koch) obtained by an enhanced TCA cycle, accumulation of sugar, and ROS detoxification. The interesting potential of Tibetan barley (*Hordeum spontaneum* C. Koch) was also described by Ahmed et al. (2013), who highlighted the drought and salt tolerance through an enhanced regulation in the glycine-betaine accumulation, in Na^+^/K^+^ ratio, sugar contents and antioxidant capacity against ROS at anthesis. Guo et al. (2016) described the potential of the calcium-sensor calcineurin B-like from *H. spontaneum* Qinghai-Tibet line (*Hs*CBL8). Rice transgenic plants overexpressing *Hs*CBL8 showed an improved salt stress tolerance by increased proline accumulation, plasma membrane protection and decrease in Na^+^ uptake.

**Figure 1:**
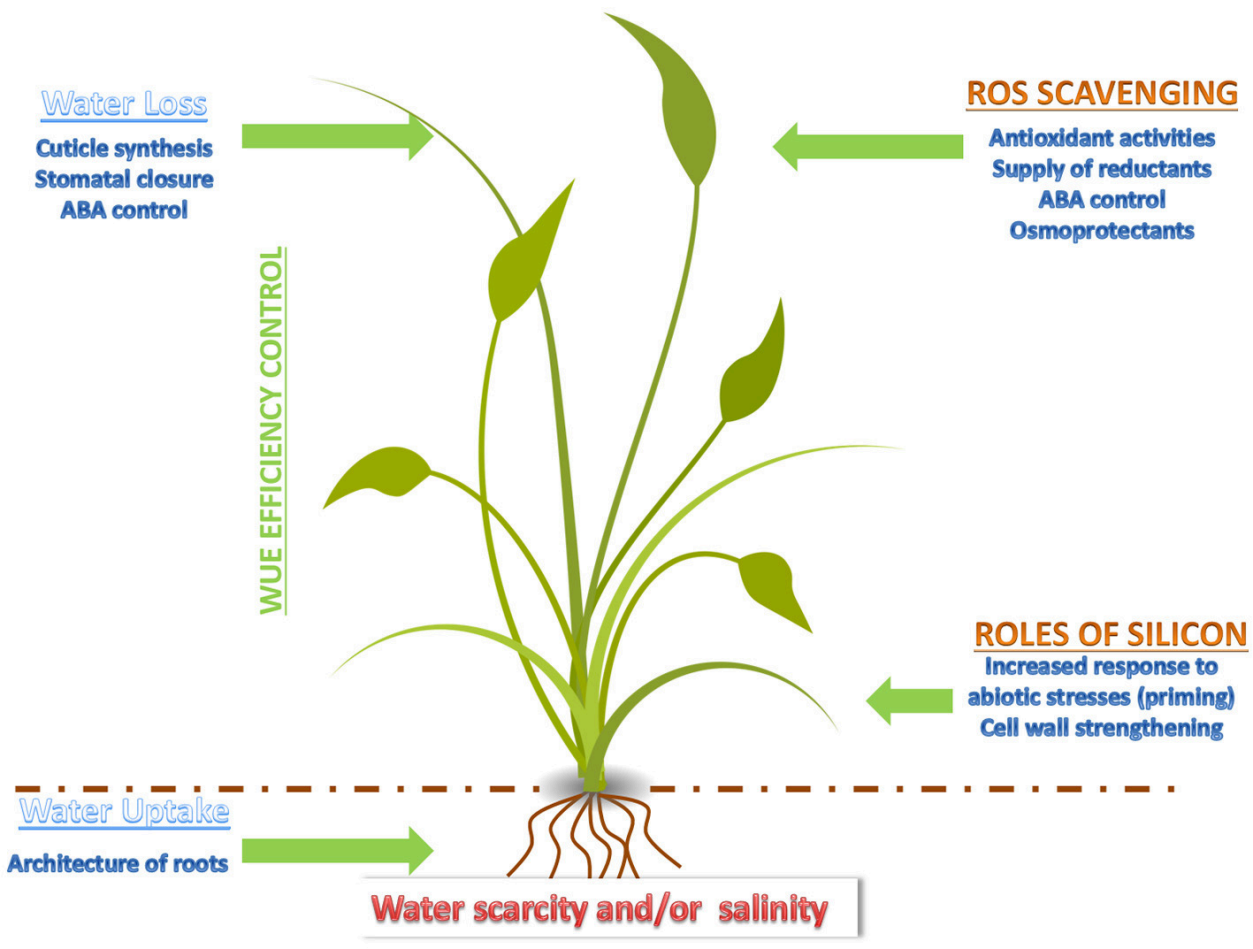
Cartoon depicting the major events and factors affecting drought and salt stress response in a monocot.

These results show that the study of landraces and wild relatives is useful to design strategies aiming at the improvement of the abiotic stress response in *Poaceae*. A schematic summary concerning the factors and key events affecting the response of monocots to drought and salt stress is presented in Figure 1. Approaches using—*omics* are, in this respect, particularly valuable, as they contribute to identify candidates and to shed light on the regulatory networks underlying the increased tolerance of *Poaceae* landraces to abiotic stresses.

## Author contributions

GG and SE conceived the idea of writing the paper. SL, JH, GG, and SE wrote the manuscript.

### Conflict of interest statement

The authors declare that the research was conducted in the absence of any commercial or financial relationships that could be construed as a potential conflict of interest.
